# Use of Essential Oils in Veterinary Medicine to Combat Bacterial and Fungal Infections

**DOI:** 10.3390/vetsci7040193

**Published:** 2020-11-30

**Authors:** Valentina Virginia Ebani, Francesca Mancianti

**Affiliations:** 1 Department of Veterinary Sciences, University of Pisa, Viale delle Piagge 2, 56124 Pisa, Italy; francesca.mancianti@unipi.it; 2 Interdepartmental Research Center “Nutraceuticals and Food for Health”, University of Pisa, Via del Borghetto 80, 56124 Pisa, Italy

**Keywords:** essential oil, bacteria, fungi, veterinary medicine

## Abstract

Essential oils (EOs) are secondary metabolites of plants employed in folk medicine for a long time thanks to their multiple properties. In the last years, their use has been introduced in veterinary medicine, too. The study of the antibacterial properties of EOs is of increasing interest, because therapies with alternative drugs are welcome to combat infections caused by antibiotic-resistant strains. Other issues could be resolved by EOs employment, such as the presence of antibiotic residues in food of animal origin and in environment. Although the in vitro antimicrobial activity of EOs has been frequently demonstrated in studies carried out on bacterial and fungal strains of different origins, there is a lack of information about their effectiveness in treating infections in animals. The scientific literature reports some studies about in vitro EOs’ activity against animal clinical bacterial and fungal isolates, but in vivo studies are very scanty. The use of EOs in therapy of companion and farm animals should follow careful studies on the toxicity of these natural products in relation to animal species and route of administration. Moreover, considering the different behavior of EOs in relation to both species and strain pathogen, before starting a therapy, an aromatogram should be executed to choose the oil with the best antimicrobial activity.

## 1. Introduction

Essential oils (EOs) are secondary metabolites of plants, obtained by distillation or mechanical processes, known since ancient times, used in folk medicine and in perfumery. Their composition varies not only on the basis of the different botanical species, but also on the part of plants used (leaves, roots, wood, bark, fruits), the season of harvest, the environmental conditions (temperature, humidity, solar irradiance, ground quality), and the extraction techniques. For these reasons, it is very hard to standardize their therapeutical employment, and therapeutical doses should be adapted when necessary. The choice of an EO to be used for clinical application should be made time after time on the basis of the different composition and consequent different activity of the oil.

However, some of these compounds show marked antimicrobial activity, which makes us consider them as promising candidates for novel drugs to treat bacterial and fungal infections.

In human medicine, aromatherapy is widely applied and an increased popularity of the use of EOs as remedia for different pathologies is reported [1,2]. In veterinary clinical practice, data concerning both the in vitro susceptibility testing and the in vivo application of EOs for treatments are not so complete such as in human medicine. So, the aim of the present study was to gather information about the antimicrobial activity of EOs to treat different bacterial and fungal affections in veterinary medicine.

The positive outcome of the in vivo trials should be correlated both to direct antimicrobial effects of EOs and/or mixtures and to their aspecific antioxidant and anti-inflammatory actions [3] along with the immunomodulatory activity [4].

In this report, we will consider mainly veterinary studies carried out from animal clinical isolates and in vivo treatment of veterinary mycoses in experimental animal models, also. There is a lack of veterinary reviews in this field, being monographic studies mostly dealing with bacterial and fungal strains from type collection cultures or, to a lesser extent, human isolates. It is not easy to compare the results obtained by the different studies due to the variability in composition of the same EOs as well as the methods employed in determining the sensitivity of the agents. However, although with some differences, it is possible to infer the suitability of some compounds as promising candidates for treating microbial affections. 

## 2. Bacterial Infections

Essential oils have been shown to have antibacterial activity with different degrees in relation to the oils and the bacteria. Deep differences have been observed among bacterial species and, for a given species, among the strains.

In bacterial pathogens, EOs primarily destabilize the cellular structure, leading to a breakdown of membrane integrity, disrupting many cellular activities including energy production and membrane transport. Furthermore, membrane rupture induced by EOs can determine the leakage of cellular components and loss of ions [4]. 

The study of the antibacterial properties of EOs is of increasing interest, because therapies with alternative drugs are welcome to combat infections caused by antibiotic-resistant strains, which represent a severe threat in human and veterinary medicine. 

The antibiotic-resistance issue is the result of overuse of antibiotics mainly in veterinary medicine as well as in animal husbandry. However, more issues are related to the employment of these drugs, such as the presence of antibiotic residues in food of animal origin (meet, milk, eggs, fish, honey) and in the environment.

Environment contamination occurs mainly through animal manure. Most antibiotics are absorbed by the body after administration and can be metabolized. Elimination of the parent drug and/or metabolites occurs essentially through the urine and/or feces. Antibiotics with poor oral absorption will exit the animal through the feces [5].

Another important veterinary route for the dissemination of antibiotic residues into the environment is through aquaculture or fish farming. In aquaculture, antibiotics are usually administered as medicated feed but also through a therapeutic bath, yielding environmental exposure through water contamination and contamination of the sediment in the pond by fish excrement and unused feed [5]. 

Antibiotic residues are a risk for the production process safety and consequently they are also an economic risk, as they inhibit biotechnological production processes involving microorganisms such as starter cultures in the dairy industry. Moreover, due to the potentially carcinogenic and toxic properties of antibiotic residues and their allergic potential, the consumption of contaminated food establishes a direct risk to public health.

In view of all these issues, EOs with antibacterial property could represent a very important alternative. The antibacterial activity of several EOs has been in vitro investigated, mainly testing reference strains. In vitro studies have been carried out assaying EOs versus isolates of humans and, less frequently, animal origin. However, the number of the studies performed to verify the in vivo usefulness of EOs to treat animal infections is very small.

### 2.1. Gram-Negative Bacteria

#### 2.1.1. Escherichia coli

*Escherichia coli* is a bacterium of the family *Enterobacteriaceae* involved in several human and animal pathologies. In veterinary medicine, it represents a severe threat because it may cause disease in all animal species determining frequent economic losses. In birds, it causes a systemic form known as Avian colibacillosis. *E. coli* infects birds, mainly poultry, by an oral–fecal cycle, even though it often affects the animals by inhalation of contaminated dust. 

The infection usually starts with septicemia followed by localized inflammation in multiple organs or sudden death [6]. Avian colibacillosis is characterized by variable lesions, among which airsacculitis and polyserositis are the most frequent. Moreover, fecal contamination of the eggs may cause *E. coli* penetration through the shell. Economic losses may be also due to slaughter waste related to necrotic cellulitis [7].

In view of the relevance of this threat, a study was carried out to verify the activity of some EOs obtained from different botanical species against *E. coli* in order to find natural products that could be employed for environment hygiene of poultry farms.

Cinnamon (*Cinnamomum zeylanicum*) and clove (*Syzygium aromaticum*) EOs evidenced good antibacterial activity, when used alone or in combination, versus *E. coli* strains isolated from poultry with colibacillosis [8].

The action of the two oils has been suggested to be related to their main compounds, eugenol and its acetate form, as suggested in other studies, too [9]. Zhang et al. [10] observed that cinnamon EO damages the permeability and integrity of *E. coli* membrane with a consequent loss of inner cell materials. A similar effect against *E. coli* was found with clove EO by Rhayour and coworkers [11] who observed damage as holes in both the cell wall and membrane.

Other EOs showed in vitro anti-*E. coli* activity. *Litsea cubeba* EO was shown to be effective against *E. coli* [8]. Li et al. [12] observed holes and gaps on the outer and inner membranes of *E. coli* cells treated with this EO, which were mainly attributed to the presence of aldehydes as geranial and neral. 

These aldheydes are the main components also of *Cymbopogon citratus* EO, which revealed good in vitro activity against *E. coli* isolates [8,13].

*Mentha piperita* EO showed high activity against *E. coli*, too [8,14]. The antimicrobial properties of peppermint EO were attributed to the major components menthol and its oxidative compound menthone [15,16]. 

Anti-*E. coli* activity was exhibited by EOs from *Ocimum basilicum* and *Pelargonium graveolens* [8,17,18]. The basil EO activity, of different degree in relation to the tested strain, has been related to its significant amount of linalool [18], as well as the high amounts of oxygenated monoterpenes in geranium EO seems to determine its antimicrobial property [8].

The application of EOs as an antibiotic alternative in swine production has been studied. Very different results have been obtained in both laboratory and field studies because of the varied product compositions, dosages, purities, and growing stages and conditions of animals. The best results were obtained with EOs mainly constituted by carvacrol, thymol, citral, eugenol, and cinnamaldehyde.

However, it was found that the minimal inhibitory concentration (MIC) of EOs needed for killing enteric pathogens may not ensure the optimal feed intake and the costs for EOs addition in feed may be too high in swine production. As a result of the lipophilic and volatile nature of EOs, there is a challenge in the effective delivery of EOs in pig gut that could partially be resolved by microencapsulation and nanotechnology. A better production performance of swine fed with EOs was observed and associated to several EOs properties that influence inflammation, oxidative stress, microbiome, gut chemosensing, and bacterial quorum sensing [19]. 

*E. coli* can affect companion animals, too, causing infection not only of the intestinal tract but also of the genitourinary system, ears, and skin. Studies to verify the usefulness of EOs administration to infected cats and dogs should be performed. The potential use of these natural products could be suggested for the topical treatment of otitis externa and dermatitis caused by antibiotic-resistant *E. coli* strains.

Multidrug-resistant isolates of *E. coli* cultured from dogs with cystitis were in vitro assayed against EOs obtained from star anise (*Illicium verum*), basil (*O. basilicum*), origanum (*Origanum vulgare*), clary sage (*Salvia sclarea*), and thymus (*Thymus vulgaris*). Even though the best results were obtained with oregano and thyme, all EOs showed anti-*E. coli* activity [20]. These oils could be proposed for the formulation of external and/or intravesical washes, although cytotoxicity and therapeutic index must be previously evaluated. 

#### 2.1.2. *Salmonella* spp.

The use of EOs to combat pathogens of the genus *Salmonella* was mainly investigated in food microbiology. In fact, foodborne infections due to *Salmonella* spp. are an increasing threat for human health; the alterations in human society with recent food processing and marketing methodology with live breeders contribute to outbreaks of salmonellosis [21].

Salmonella strains resistant to commercial antibiotics have emerged as a great health concern to the consumers. Moreover, strains of this genus are the cause of disease in farm animals and pets with consequent economic losses and risk of transmission to humans. 

The oils R (+)-limonene, orange terpenes, cold compressed orange oil, trans-cinnamaldehyde, and carvacrol were tested against fifteen different *Salmonella enterica* serovar Heidelberg strains isolated from multiple sources such as turkey, poultry and poultry egg houses, cattle, swine, and humans. Carvacrol and trans-cinnamaldehyde completely inhibited the growth of all *S.* Heidelberg isolates, while R (+)-limonene and orange terpenes did not show any inhibitory activity against the same strains; cold compressed orange oil only inhibited the growth of two isolates [22]. 

EOs obtained from *Aloysia triphylla*, *C. zeylanicum*, *C. citratus*, *L. cubeba*, *M. piperita*, and *S. aromaticum* have been tested against *S. enterica* serovar Enteritidis and *S. enterica* serovar Thyphimurium strains isolated from poultry. These serovars are important zoonotic pathogens associated to severe diseases in persons, birds, and mammals. *C. zeylanicum* showed the highest anti-*Salmonella* activity, followed by *S. aromaticum* EO. The use of these EOs in poultry diet, combined with *Saccharomyces cerevisiae* administration, has been suggested for an integrated approach to avoid *Salmonella* intestinal colonization. Moreover, EOs obtained from *C. zeylanicum* and *S. aromaticum* could be employed, alone or in combination, in a farm environment for disinfection [23]. 

The antimicrobial activity of these EOs has been attributed to their main components: cinnamaldehyde, present in *C. zeylanicum* EO, and eugenol present in *C. zeylanicum* as well as in *S. aromaticum.* These substances react with lipid and hydroxyl radicals converting them into stable products through their hydrogen-donating ability [24]. Moreover, the two components are able to inhibit the production of essential enzymes by the bacteria due to the presence of a carbonyl group that binds and inactivates them and/or causes damage to the cell wall of the bacteria [25]. 

The use of EOs in poultry nutrition is the object of interest, because, other than the potential antimicrobial activity of EOs, it seems to improve the quality of meet and egg production [26].

The in vivo effects of EOs combined with organic acids (EOA) on *Salmonella enterica* serovar. Enteritidis challenged chickens were studied by Zhang et al. [27]. Two types of mixtures were assayed. EOA1 was mainly composed by thymol, carvacrol, benzoic acid, butyric acid, and excipients such as silicon dioxide and palm oil. The main components of EOA2 were cinnamylaldehyde, caproic acid, benzoic acid, butyric acid, and the excipients silicon dioxide and palm oil. Both mixtures showed bacteriostatic activity against *S.* Enteritidis in the treated animals, even though EOA1 was more effective. Furthermore, both mixtures reduced the contents of Tumor necrosis factor-alpha in the chickens treated with them. 

The antimicrobial and antioxidant properties of EOs and organic acids also have been reported by other authors [28,29]. Thanks to these features, EOA seem to improve animal intestinal health and promote the absorption of nutrients; thus, they could be largely used as antibiotic substitutes. 

#### 2.1.3. *Pseudomonas* spp.

*Pseudomonas aeruginosa* is a common cause of infections in hospitalized patients, animals, and humans, inducing bacteremia and pneumonia as well as urinary, genital tract, and skin infections. Other members of *Pseudomonas* genus are involved in infections of different body districts albeit less frequently. 

*P. aeruginosa* has developed resistance to many types of antibiotics. In particular, intrinsic resistance to a wide range of antibiotics is due to the nature of the outer membrane of this pathogen that protects the bacterium against toxic agents and enables it to form a biofilm. The occurrence of extended-spectrum β-lactamases in *P. aeruginosa* has also resulted in the lack of specific antibiotics for this microorganism [30,31].

Essential extracts from the bark of *Cinnamomum* spp. plants have been reported to possess antibacterial activity toward a range of microbes, including *P. aeruginosa* [32,33]. 

Kavanaugh et al. [34] studied the ability of some EOs, including *Cinnamomum*, to inhibit the growth of this pathogen and the formation of biofilm. Cassia (*Cinnamomum aromaticum*), clove (*S. aromaticum*), Peru balsam (*Myroxylon balsamum*), red thyme (*T. vulgaris*), and tea tree (*Melaleuca alternifolia*) oils were effective in killing *P. aeruginosa* in vitro. Moreover, cassia, red thyme, and Peru balsam EOs were active against the biofilm formation, too.

*T. vulgaris* EO was shown to be effective against *P. aeruginosa* in other studies where it inhibited the in vitro growth of human clinical multidrug-resistant isolates [35].

However, thyme EOs did not always show activity versus this pathogen. For example, not promising results were obtained assaying a *P. aeruginosa* strain that was isolated from a dog with externa otitis and was shown to be resistant to several antibiotics, versus commercial EOs including *T. vulgaris* oil. The canine strain was also tested with oils obtained from roman chamomile (*Anthemis nobilis*), star anise (*I. verum*), lavender (*Lavandula hybrida*), litsea (*L. cubeba*), basil (*O. basilicum*), oregano (*O. vulgare* subsp. *hirticum*), rosemary (*Rosmarinus officinalis*), and clary sage (*S. sclarea*). Scarce activity was shown only by *S. sclarea*, *O. basilicum*, and *R. officinalis*, whereas the remaining EOs, including thyme, have no activity [36].

EOs obtained from cinnamon bark (*C. zeylanicum*), lemongrass (*Cymbopogon flexuosus*), and clove (*S. aromaticum*) were examined on 20 human clinical multidrug isolates of *P. aeruginosa*. Cinnamon bark oil showed the strongest activity against all followed by lemongrass oil and clove oil. Furthermore, promising results were obtained when cinnamon oil was associated to cinnamaldehyde; in fact, higher bactericidal activity was observed against all isolates [37].

*Pinus eldarica* bark EO was active against a reference strain of *P. aeruginosa* [38].

The effects of basil (*O. basilicum*) and sage (*Salvia officinalis*) EOs on selected virulence factors (biofilm formation, mature biofilm resistance, motility, and pyocyanin production) of *P. aeruginosa* human clinical isolates were recently evaluated by Pejicic et al. [39]. Interesting results were obtained; in fact, both oils inhibited biofilm formation and significant reductions were also noted when the oils were applied to mature biofilms. Likewise, pyocyanin production, swimming, swarming, and twitching motility patterns were highly affected by basil and sage oils. The anti-*P. aeruginosa* properties of the two EOs seem to be associated to their main components: linalool and (*E*)-anethole in the investigated basil oil, α-thujone, and camphor in the studied sage oil [39]. 

Kacaniova et al. [40] carried out a study of veterinary interest. They assayed the antimicrobial activity of several EOs against ten different *Pseudomonas* strains cultured from freshly caught greshwater fish. All isolates, belonging to the species *P. agglomerans*, *P. antarctica*, *P. brassicacearum*, *P. frederiksbergensis*, *P. koreensis*, *P. lundensis*, *P. mandelii*, *P. proteolytica*, *P. synxantha*, and *P. veronii*, were tested against 21 EOs: *L. angustifolia*, *C. zeylanicum*, *Pinus montana*, *M. piperita*, *Foeniculum vulgare*, *Pinus sylvestris*, *Satureja hortensis*, *O. vulgare*, *Pimpinella anisum*, *R. officinalis*, *S. officinalis*, *Abies alba*, *Citrus aurantium* var. *dulce*, *Citrus sinensis*, *Cymbopogon nardus*, *Mentha spicata* var. *crispa*, *T. vulgaris*, *Carum carvi*, *Thymus serpyllum*, *O. basilicum*, and *Coriandrum sativum*. All tested EOs exhibited antimicrobial activity even though *C. zeylanicum* EO was the most effective against *Pseudomonas* spp. both according to the disc diffusion and MIC methods. EOs from C. *nardus*, *O. vulgare*, *F. vulgare*, and *T. serpyllum* showed the highest antioxidant activity. This investigation gave data that was useful for finding alternative treatment against bacterial species frequently isolated from the skin, gills, and intestine of fish and determining the spoilage processes of freshly caught and processed fish [41].

#### 2.1.4. *Campylobacter* spp.

Bacteria of the genus *Campylobacter* are pathogens responsible for diseases in humans and several animal species. It is considered a leading cause of human gastroenteritis worldwide. In fact, it was ranked 3rd as the leading impact of public health due to foodborne illness behind non-typhoidal *Salmonella* and *Toxoplasma* [42].

*C. jejuni* is the species most widespread, but other species such as *C. coli*, *C. lari*, and *C. upsaliensis* are frequently isolated from animal and human clinical specimens.

Broilers and layer flocks have consistently been shown to contain very high *Campylobacter* prevalences. Through contamination of the poultry carcass, *Campylobacter* can cause foodborne illness and has been the etiological agent in outbreaks caused by poultry products.

Considering that EOs seem to improve the immune system of poultry, their use is a promising factor to combat *Campylobacter* infection, too. In fact, *Campylobacter* colonization may elicit an immune response, and therefore, there is potential to reduce *Campylobacter* concentrations through immune system modulation [43]. EOs may also interact with *Campylobacter* populations directly. 

Several studies demonstrated the in vitro activity of different EOs against *Campylobacter* strains, but fewer information about the in vivo properties of the same oils are available in the literature.

As it occurs when EOs are employed against other bacteria, the EOs effectiveness versus *Campylobacter* isolates is related to the EO because each of them has a different chemical composition and mechanism of action.

The antibacterial activity of numerous EOs was assayed by Friedman et al. [44] testing isolates from food and clinical sources. The oils most active against *C. jejuni* were marigold, ginger root, jasmine, patchouli, gardenia, cedarwood, carrot seed, celery seed, mugwort, spikenard, and orange bitter oils. 

Tea tree oil, lemon myrtle oil, and *Leptospermum* oil were shown to be active against *C. jejuni* and *C. coli* of poultry origin [45], as well as *Origanum syriacum* against a *C. jejuni* reference strain [46].

On the basis of the results obtained in the different investigations, active EOs are those containing some compounds; in particular, the components most active against *C. jejuni* were shown to be cinnamaldehyde, estragole, carvacrol, benzaldehyde, citral, thymol, eugenol, perillaldehyde, carvone R, and geranyl acetate [44].

Mammals can be infected by *Campylobacter* spp., too. In particular, dogs and cats may develop gastroenteric clinical signs or be asymptomatic, but in all cases, they are a potential source of infections for their owners. Studies to evaluate the effect of EOs versus *Campylobacter* strains isolated from pets are not available in the literature; however, it can be supposed that canine and feline isolates may be sensitive to EOs shown to be active against *Campylobacter* strains of different origin. It might be interesting to evaluate if in these animal species, in vitro effective EOs could be employed in alternative to conventional drugs or in combination with them to enhance the antibacterial activity and/or improve the immune system similarly to what happens in poultry.

### 2.2. Gram-Positive Bacteria

#### 2.2.1. *Staphylococcus* spp.

Members of genus *Staphylococcus* are well-known opportunistic pathogens of humans, but they represent a severe concern in veterinary medicine, too. In fact, they cause infections of different anatomical districts in mammals, birds, and cold-blooded animals.

The genus includes coagulase-positive and coagulase-negative staphylococci, which are both involved in infections of different severity degrees. Among the coagulase-positive, *S. aureus* is the most known species. It is responsible for a wide variety of human infections that include mild superficial skin infections, osteomyelitis, implant-associated heart valve, native valve endocarditis, severe sepsis, and bacteremia. It causes similar pathologies in animals. 

*S. aureus* has been isolated from several body sites of dogs and cats, mainly skin, anterior nares, and the anal region. Furthermore, companion animals are frequently infected by *S. pseudointermedius*, which is part of the microflora of the upper respiratory tract, mucosal, and skin surfaces, but it may become an invasive pathogen determining different pathologies. However, other species, such as *S. chromogenes*, *S. xylosus*, and *S. hyicus*, can infect companion animals.

Staphylococcal infections may represent severe economic concern in farm animals, too.

Several studies have demonstrated the anti-staphylococcal activity of *O. vulgare* and *T. vulgaris* EOs thanks to their main components carvacrol and thymol [36,47,48,49,50]. However, EOs obtained also from other botanical species were shown to be effective against staphylococci.

*Satureja montana* EO exhibited good anti-staphylococcal activity [36]. The antimicrobial effectiveness of *S. montana* EO against some Gram-positive and Gram-negative bacteria was previously observed, and it was related to major compounds, such as carvacrol. In particular, Vitanza et al. [51] found that *S. aureus*, submitted to the action of *S. montana* EO, showed collapse of cell walls without breaks.

Ebani and coworkers [50] found that EOs from *Commiphora myrrha* and *C. zeylanicum* had low activity against some canine isolates of the species *S. aureus*, *S. pseudointermedius*, *S. chromogenes*, and *S. hyicus*, whereas no activity was observed against *S. xylosus*. Similarly, Adam and Selim [52] observed a slight sensitivity of *S. aureus* to myrrh oil. Conversely, Mahboubi and Kazempour [53] found relevant activity of *C. myrrha* against a *S. aureus* ATCC strain. 

*L. cubeba* was demonstrated to be an effective bacterial inhibitor and bactericide against some methicillin-resistant *S. aureus* strains for a destructive effect on the bacterial cell membrane [54]. EO from this plant did not always show good anti-staphylococcal activity, as demonstrated when it was tested against clinical canine isolates of different species [50].

Canine clinical isolates of *S. aureus* and *S. pseudointermedius* were shown to be quite sensitive also to *Helichrysum pandurifolium*, *H. trilineatum*, and *H. araxinum* [55,56].

The in vitro antimicrobial activity of some EOs was tested against four methicillin-resistant *S. pseudintermedius* and four methicillin-susceptible *S. pseudintermedius* canine cutaneous clinical isolates. The best results against all strains were provided by *C. zeylanicum*, *M. officinalis*, *Leptospermum scoparium*, *S. montana*, and *C. citratus* EOs [57].

*S. aureus* was shown to be sensitive in some studies to lemongrass *C. citratus* and lemon balm *M. officinalis* EOs [58,59]. The growth of *S. aureus* reference strains was inhibited by *A. triphylla* EO, too [60,61,62], which compromises the structural integrity of the plasmic membrane and induces a loss of the cytoplasmic contents, with consequent cellular death [62].

Bovine mastitis caused by *S. aureus* is a great concern in veterinary medicine for the high economic impact. Abboud et al. [63] observed in an in vivo study a strong antibacterial activity of essential oils of *T. vulgaris* and *L. angustifolia* against both *Staphylococcus* sp. and *Streptococcus* sp. An intramammary application of these oils and of a mixture of them caused a drastic decrease in bacterial count in the different samples of milk after 4 consecutive days of treatments. In the same study, the stronger antibacterial activity was achieved by the external application of these oils in vaseline with a rate of recovery of 100% with thymus essential oils.

#### 2.2.2. *Streptococcus* spp.

Data reported in the literature are mainly concerning the EOs activity against *Streptococcus* spp. strains of human origin. Even though some species, as *S. pyogenes*, are able to infect both humans and animals, other streptococcal species are mainly a cause of infection and diseases in several farm and companion animals.

*Cymbopogon citratus* EO exhibited an inhibitory effect on streptococcal strains of human oral origin [64].

Previous studies found that also *C. zeylanicum* EO was able to inhibit human streptococcal isolates. In particular, *C. zeylanicum* was shown to be active against *Streptococcus agalactiae* strains isolated from the anus and vagina of pregnant women [65] and against *Streptococcus mutans* isolated from patients with oral infections [66]. Antimicrobial activity against several pathogens, including some *Streptococcus* species, was observed testing *Cinnamomum verum* and *Cinnamomum cassia* EOs [67,68,69].

The antistreptococcal activity of cinnamon EO has been related to its main compound cinnamaldehyde, as it occurs against other bacteria. 

*C. verum* was shown to be in vitro and in vivo effective against *Streptococcus iniae*, which is a pathogen of fish. During an in vivo trial, no mortality was apparent in tilapia fed on fish diets supplemented with 0.4% (*w*/*w*) of cinnamon oil and with 0.1% (*w*/*w*) of oxytetracycline 5 days prior to infection with *S. iniae* [69].

The modulating effect of dietary with clove (*S. aromaticum*) EO on the antioxidant and immunological status of Nile tilapia following *Streptococcus iniae* infection was evaluated in an in vivo experiment. Clove EO stimulated the fish immunity against bacterial challenge. Moreover, the antibacterial effect of this oil was shown to be partly mediated by inducing hepatic hepcidin expression [70]. 

*S. aromaticum* showed good activity against *S. mutans*, too, and eugenol was identified as the main compound that influences the antimicrobial activity [71,72].

*O*. *vulgare* and *T. vulgaris* EOs exhibited antistreptococcal activity against human isolates, mainly *S. mutans* and *S. pyogenes* [66,73]. 

A study of veterinary interest was carried out by de Aguiar et al. [74], according to which thyme and oregano EOs were in vitro effective against *Streptococcus suis* isolates. 

#### 2.2.3. *Enterococcus* spp.

Enterococci belong to the gastrointestinal microflora of animals and humans and are widespread in terrestrial and water habitats [75]. Enterococci bring numerous benefits in the gastrointestinal tract, mainly the probiotic activity and bacteriocins production. However, enterococci are well known for their pathogen role, too. In fact, they are opportunistic bacteria able to cause severe human and animal infections. Furthermore, they have a strong ability to acquire, express, and transfer antimicrobial resistance [75,76]. 

*E. faecalis* and *E. faecium* are the species most frequently isolated in urinary tract infections, endocarditis, wound infections, sepsis, and neonatal infections [77,78]. *Enterococcus* spp. are also a cause of mastitis in cattle, diarrhea in swine and cattle, as well as endocarditis, septicemia, spondylitis, and amyloid arthropathy in poultry [79,80]. 

Data about the effectiveness of EOs against *Enterococcus* spp. isolates of animal origin are very scanty.

A study was carried out on *Enterococcus* spp. strains isolated from dogs and cats with severe cases of urinary tract infections that were shown to be not sensitive (resistant and intermediate) to several antibiotics, in order to verify the in vitro sensitivity to some commercial EOs. *O. vulgare* and *T. vulgaris* showed good activity. The same strains were shown to be resistant to *I. verum* and *S. sclarea*, which instead were moderately effective against *E. coli* urinary isolates [20]. This different behavior has been related to the dissimilar structures of the Gram-positive and Gram-negative bacteria cell walls, as also supposed by Benmalek et al. [81] who found similar results when tested *I. verum* EO against *E. coli* and *S. aureus*. The activity of *I. verum* against enterococci is scantly investigated; however, Hawrelak et al. [82] found a very weak effectiveness of this EO against *E. faecalis*. 

Conversely, *S. officinalis* EO was found active against both Gram-negative bacteria, such as *E. coli*, and Gram-positive bacteria including enterococci [83]. 

Thyme oil was shown to be able to inhibit the biofilm formation of enterococci by affecting cell adherence and exopolysaccharide synthesis [84]. This property could be useful as an effective green antibacterial agent in food processing, but it is important in the treatment of infections, too, because the biofilm formation influences the enterococcal infection pathogenesis.

EOs that were shown to be in vitro effective against *Enterococcus* spp. could be employed in veterinary medicine as a natural alternative to antibiotics in local treatment such as external or intravesical washes in cases of urinary infections, or external application in cases of infection of skin and otitis externa.

Instead, an oral administration should be avoided or limited, because EOs could negatively influence the natural presence of enterococci in the intestinal microflora, decreasing the probiotic effect of these bacteria.

### 2.3. Mycobacterium spp.

Even though some studies were carried out to verify the EOs’ activity against *Mycobacterium tuberculosis* and *M. bovis*, which are species responsible for tuberculosis in humans and animals [85,86], the employment of alternative treatments against tuberculosis are not welcome in view of the severity of this infectious disease.

On the other hand, EOs could represent alternative natural remedies to combat infections caused by non-tuberculous mycobacteria (NTM).

These are opportunistic bacteria that are widespread in the environment, being present in soil and water. They are usually transmitted by aerosol inhalation, the ingestion of contaminated food and water, and inoculation through the skin. Several NTM strains produce biofilm, becoming more resistant to environmental stresses, disinfectant, and antimicrobial drugs [87]. NTM are responsible for different forms in humans, such as lymphadenitis, skin infections, pulmonary mycobacteriosis, and disseminated forms. These mycobacteria are important in veterinary medicine, too; in fact, they cause similar infection in several animal species. Cutaneous forms in dogs and cats are often related to NTM species. 

Therapy with conventional drugs is used, but it is not always successful. For this reason, local treatment with EOs could be a promising alternative, but studies about the in vivo activity of EOs other than their no citotoxicity are necessary.

*M. avium avium* is classified as a NTM agent, although it is related to tuberculosis in the avian population.

The antibacterial and antibiofilm potential of *Juniperus communis*, *Helichrysum italicum,* and *L. hybrida* EOs against NTM, in particular *M. avium avium*, *M. intracellulare,* and *M. gordonae*, was in vitro demonstrated [87,88].

EO from *Zingiber officinale*, characterized by a high number of monoterpenes and sesquiterpenes, showed activity against some NTM species, mainly *M. chelonae* and *M. abscessus* subsp. *massiliense* [86].

Studies have been performed to verify the in vitro activity of some EOs versus *M. avium* subsp. *paratuberculosis*, which is a NTM pathogen causing paratuberculosis, a severe disease in ruminants with relevant economic losses. Cinnamon and oregano oils showed good activity [89,90], whereas *Myrtus communis* EO exhibited weak effectiveness [91].

*M. avium paratuberculosis* is a pathogen with localization in enteric tract and udders. It is difficult to suppose a therapy with EOs to treat ruminants affected by paratuberculosis, but natural products, which have been shown to be in vitro effective, could be used to reduce the environmental farm contamination by this pathogen.

Table 1 lists the EOs reported as active against bacterial strains of animal origin during in vitro and in vivo studies.

## 3. Fungal Infections

Mycoses account among the more frequently observed infections in veterinary practice. Most systemic antimycotic drugs are used off label in pets; furthermore, the administration in food-producing animals is forbidden. Therefore, their withdrawal period is not reported. For these reasons, alternative solutions to control mycotic diseases would be welcome.

Among the superficial mycoses of both pets and livestock, the dermatophytoses are widely spread and have a large impact on One Health when zoonotic agents are involved. Furthermore, these infections can negatively affect animal welfare as well as productions [92,93].

### 3.1. Dermatophytes

Dermatophytes are keratinophilic and keratinolytic molds that are responsible for ringworm and dermatophytic pseudomycetoma. These fungal affections can be treated topically or by systemic antimycotic drugs, which are needed when lesions are diffuse. However, itraconazole is the only veterinary licensed drug for dermatophyte treatment. Moreover, it should be employed for feline microsporiasis, only. For these reasons, all other therapeutic systemic options are used off label, and veterinary therapeutic arsenal is very limited. Furthermore, the observation of emerging azole resistance in field strains [94] suggests the usefulness of searching for alternative molecules for the treatment.

EOs have been checked against dermatophytes, both by in vitro testing and in clinical or preclinical trials.

*Microsporum canis* has been widely studied. This is a zoophilic, zoonotic dermatophyte responsible for ringworm, kerion, and dermatophytic pseudomycetoma in mammals and for *tinea capitis* and *tinea corporis* in human patients, mostly in children. Cats are the reservoir animal species and are essential in spreading the agent to other animals and human beings, as well as into the environment. This dermatophyte can be easily found, cultured, and maintained in the laboratory. For these reasons, studies dealing with *M. canis* sensitivity are the most numerous, involving the human medicine, also. 

Other dermatophytes involved in animal infections are the zoonotic species *Trichophyton mentagrophytes* and, to a lesser extent, *Trichophyton erinacei.* These fungi have as biologic reservoir rodents and hedgehogs, respectively, and they are responsible for human infection, with a wide range of clinical pictures (*tinea corporis*, *tinea capitis*, *tinea barbae*). Another important zoonotic agent is *Trichophyton verrucosum*, which is responsible, in human patients, for the same clinical forms caused by *T. mentagrophytes*, following occupational exposure to infected calves. However, probably due to the difficulty of the in vitro cultivation of this dermatophyte, data about its sensitivity to natural products are lacking.

*Microsporum gypseum* is a geophilic complex of species that is responsible for animal dermatomycosis and mostly for *tinea corporis* in human beings. The infection occurs following contact with soil, where these fungi proliferate on keratin from hair baits shed by humans and animals.

There are many in vitro studies on clinical veterinary specimens of dermatophytes, comprehending several EOs, which show different antifungal activity.

To the best of our knowledge, the first veterinary paper goes back to 1994 [95] and deals with EOs derived by botanical species, belonging to the Family *Lamiaceae*. *M. canis* and *M. gypseum* cultured from symptomatic cats, dogs, and horses, checked by a macrodilution test, showed a marked sensitivity to EOs obtained from *T. vulgaris* and *S. montana*. *Calamintha nepeta* exhibited a moderate antifungal activity, while *L. angustifolia*, and *R. officinalis* had a weak effectiveness, varying among the different fungal isolates. Accordingly, *Clinopodium (Calamintha) nepeta* was recently found to be moderately active against *M. canis* and *M. gypseum*, but the EO obtained from a strain of subspecies *glandulosum* rich in piperitenone oxyde (39.3%) revealed an unexpected effectiveness against *T. mentagrophytes* [96], while all the dermatophytes were shown to be very sensitive to *Thymus numidicus* [97]. The poor antidermatophytic activity of *R. officinalis* (mostly containing alfa pinene, 1.8 cineol, and camphor) was confirmed by other authors [98,99,100]. Otherwise, this EO would strongly enhance the activity of *T. serpillum* and *O. vulgare*, with a synergystic effect in a mixture (5%, 2% and 5%, respectively) against *M. canis* and *T. mentagrophytes*, both in vitro and in vivo [98,99]. Moreover, *R. officinalis* is suitable for an in vivo administration for its anti-inflammatory action [101]. 

The positive results yielded by *Thymus* and *Satureja* are probably due to their high contents of thymol and carvacrol, whose fungicidal activity would be due to the inhibition of ergosterol biosynthesis and disruption of the membrane [102,103]. In addition, *T. serpyllum*, such as *O. vulgare* (rich in carvacrol), scored active in vitro against *M. canis* [98], and several dermatophytes (*M. canis*, *T. mentagrophytes*, *T. erinacei*, *Trichophyton terrestre,* and *M. gypseum*). In particular, *Trichophyton* species, would show a higher sensitivity versus thymol when compared to *Microsporum* spp. [100]. In fact, ovine strains of *T. mentagrophytes* were shown to be highly sensitive to 0.0625% carvacrol versus 0.125% thymol [99], suggesting a slight degree of variability among the different fungal isolates. Thymol was the main compound (59.65%) contained in *Lippia sidoides* EO, which appeared quite active in an agar well diffusion test against feline and canine isolates of *M. canis* [104].

Both thymol and carvacrol exert toxic effects on both animals and human cells [105,106]. For these reasons, other botanical species can be usefully employed. EO from *Croton argyrophylloides*, containing as main components spathulenol and bicyclogermacrene, showed a good activity against *M. canis*, in comparison with other *Croton* species [107].

*L. cubeba* EO appears as another interesting alternative, showing MIC values near *O. vulgare*. Its efficacy was probably due to the high contents of geranial and neral, which was proven to be strongly effective for their fungistatic and fungicidal activities against most dermatophytic species, at low concentrations, such as 0.1% for *M. canis* and 0.25% for *M. gypseum* and *Trichophyton* species. EO from *Foeniculum vulgare*, a botanical species belonging to the *Apiaceae* Family, is rich in anethol and fenchone. This latter is considered to have an antidermatophyte activity [108] and was moderately effective against *M. canis* and *M. gypseum.* These fungal species were shown to be in fact sensitive to fenchone only (0.25% and 0.5%) [100].

Geraniol is an oxygenate monotherpene with an antidermatophyte effect [109] due to its ability to bind ergosterol [110], although after a study on the permeability of the plasma membrane in *Candida albicans*, Leite et al. [111] found that ergosterol is not a direct target of geraniol. It has MICs ranging from 0.25 to 1%, against dermatophytes [100] and is contained in EOs from different botanical species. *P. graveolens* EO, i.e., despite its content of geraniol, does not exert a strong antidermatophyte activity [100], while *C. nardus* EO has shown a marked inhibitory activity versus *M. canis* [112]. Soares et al. [113], using a *Coriandrum sativum* EO, report MICs and MFCs ranging from 78 to 620 and 150 to 1250 μg/mL, respectively, indicating a wide variability among the feline isolates of *M. canis* essayed. 

EOs from *Citrus* fruits would mostly show a negligeable antidermatophyte activity. In detail, *Citrus limon* EO was proven neither effective against *M. canis* [98] nor other dermatophytes [99], as well as its variety Pompia [114] and *Citrus medica* and *Citrus bergamia* [100]. Only one out of three cultivars of *C. bergamia* (*C. bergamia* v. *fantastico*), and *Citrus japonica* EOs would show a moderate activity (0.5%) versus *M. canis*, *M. gypseum,* and *T. mentagrophytes*, while *Citrus × myrtifolia* did not exert any antimycotic effect when tested at 10% [115]. *Citrus* spp. mostly contains large amounts of limonene, which would have a negligible action against *M. canis* with an MIC > 10% [100], although Chee et al. [116] refer a fungicidal activity at 0.5% against the anthropophilic *Trichophyton rubrum.*

All these features cannot be only explained by the different sensitivities of the fungal isolates, which are striking mostly for yeasts in respect to molds, but also by the synergistic and/or antagonistic effects of the single components of EOs, and the activity of the phytocomplex has to be taken into account. In fact, the effects of the main actives derived from essential oils may vary depending on whether they are isolated or included in a mixture [117].

Tea tree (*M. alternifolia*) oil has been widely used in Australian folk medicine and had shown a moderate activity on *Trichophyton equinum*, the etiologic agent of equine ringworm both in vivo [118] and in vitro [119]. 

Other EOs, such *Salvia dolomitica* and *Salvia somalensis,* have been in vitro checked and shown to have poor antidermatophye effects [120].

*Helichrysum* spp. are plants largely employed in folk medicine [121,122]. Veterinary studies showed striking differences of antidermatophyte activity among the different species. Feline clinical isolates of *M. canis* and *T. mentagrophytes* were shown to be susceptible to *H. pandurifolium* EO (0.25% and 0.5%, respectively), to *H*. *edwardsii* (0.25% and 1%), and to *H cooperi* (0.5% and 1%), while *H. odoratissimum*, *and H. trilineatum* were less effective [55], and *H. italicum* and *H. araxinum* appeared quite uneffective [56,100].

The chemical composition was checked, revealing that *H. pandurifolium* was rich in viridiflor and contains beta pinene, spathulenol, delta cadinene, and epi-alfa-muurolol [123,124,125,126], which probably develop a synergistic action. The effectiveness of *H. cooperi* would be due to the presence of a good amount of terpinen-4-ol [127] and himachalol [128], while the richness in tujone of *H. edwardsii* could explain its ability in inhibiting dermatophytes [55].

Only a few studies deal with the in vivo testing both on naturally infected animals and on experimental models.

In detail, tea tree EO 25% in sweet almond oil was administered to 30 horses affected by ringworm caused by *T. equinum* twice a day for 15 days, yielding a complete etiologic and clinical healing [118].

A mixture composed by *T. serpyllum* (2%), *O. vulgare* (5%), and *R. officinalis* (5%) in sweet almond oil was administered twice a day for a month to seven cats infected by *M. canis* and allowed a complete etiologic and clinical healing in four of them [98]. This remedium was also successfully administered twice daily for 15 days to 13 sheep affected by dermatophytosis caused by *T. mentagrophytes* [99]. The same mixture was also in vivo applied as a shampoo formulation by twice a week washing instead of a conventional antimycotic shampoo for the local treatment of feline microsporiasis in seven naturally infected cats treated with systemic itraconazole. All these subjects healed at the 11th week after the start of the treatment [129].

Experimental ringworm in Guinea pig was successfully treated with a base of Vaseline^®^ petroleum jelly containing 10% eugenol and neridol from japanese cypress oil, which was daily applied topically to the skin lesion infected with *M. gypseum* for 3 weeks [130]. Neridol was able to improve the clinical picture, and its application, together with eugenol, suggests an early remission of lesions. *M. piperita* EO appeared active in ointments containing 1% EO and menthol mixed against a rat model of experimentally induced dermatophytosis by anthropophilic *Trichophyton* spp [131]. Finally, *Cymbopogon martini* (rich in transgeraniol) and *Chenopodium ambrosioides* EOs were in vitro tested with very low MICs. These compounds, administered as ointment 1% in jelly petroleum twice a day for 21 days were effective against *M. gypseum*-induced ringworm in a guinea pig model [132].

### 3.2. Malassezia pachydermatis

*Malassezia pachydermatis* yeasts are part of the cutaneous microbiota of mammals and would act as the main fungal organisms responsible for otitis and seborrhoic dermatitis in atopic dogs. Skin disease is favored by folds, atopia, endocrinopathies, keratine defects, and in cats by some visceral paraneoplastic syndromes [133]. Furthermore, a zoonotic role of this lipophilic yeast has been reported time after time [134,135,136]. 

Different EOs have been assayed in in vitro studies.

*M. alternifolia* EOs has been observed strongly active (with MIC values ranging from 0.06 to 0.13%) against canine isolates from dermatologic samples [137]. The EO was effective versus yeast from otologic specimens [138], and a synergistic action with clotrimazole was then reported [139].

Thymol and carvacrol appeared strongly effective [140,141]. These findings would elucidate the excellent inhibitory activity of *Zataria multiflora* [140], *Thymus* spp. [140,141,142], *Satureja* spp. [50,143], and *O. vulgare* [141,142,143]. This latter was proven to exert a synergistic action with clotrimazole also [139]. 

*Cymbopogon* spp. EOs were shown to be active against *M. pachydermatis* [143]. However, in another recent study, five dermatologic yeast isolates tested not sensitive to *T. vulgaris* and *O. vulgare*, as well as to *C. citratus* [50], suggesting a wide variability among the clinical isolates of the yeast [143].

*C. limon* EO scored active at 1% such as *M. piperita*, [142]. This latter was able to show a synergistic effect with clotrimazole [139].

Cynnamon was reported to have a good activity [143,144], which was probably due to cinnamaldehyde [143]; this was partially confirmed by Ebani et al. [50], who report a strong inhibitory effect of *A. triphylla* EO, rich in limonene and sabinene, which is able to inhibit the yeast at dilutions ranging from 0.5 to 0.75%.

Beta-thujaplicin, largely contained in *Chamaecyparis obtusa* EO [145], showed an anti-Malassezia effect and was recommended as a remedy for canine otitis externa due to its antifungal, anti-inflammatory, and deodorant effects [146].

Considering the commensal features of this yeast, the use of EOs is very suitable, mostly in vivo, to inhibit the fungal overgrowth and to correct some predisposing factors.

Atopic dogs affected by recurrent dermatitis were successfully treated with a mixture composed by *Citrus aurantium* and *Lavandula officinalis* 1%, with *O. vulgare*, *Origanum majorana*, *M. piperita*, and *H. italicum* 0.5%, which was administered twice daily for a month without any relapse after the end of the therapy [147].

Malassezia otitis was treated with five different mixtures of EOs administered once daily for two weeks. The remedium composed by *C. limon* and *R. officinalis* 1% with *Salvia sclarea* and *Anthemis nobilis* 0.5% yielded excellent results in all treated dogs. The other mixtures composed by *C. paradisi*, *S. sclarea*, and *Ocimum basilicum* 0.5% with *R. officinalis* 1%, and by *S. sclarea*, *R. officinalis*, *Lavandula hybrida* 1% showed good efficacy also. The mixture constituted by *C. limon* and *C. paradisi* 1% with *R. officinalis* and *A. nobilis* 0.5% yielded adverse effects. Finally, *A. nobilis* and *L. hybrida* 1%, with *C. paradisi* and *T. vulgaris* 0.5% was well tolerated but less effective when compared to the others. However, the number of the blastospores was not influenced by any treatments, suggesting a global effect on the ear micro-habitat due to the antiflogistic and eudermic activity of some of selected EOs [148].

### 3.3. Aspergillus spp.

*Aspergillus* spp. are environmental molds, occasionally involved in bovine mycotic abortion, in canine otitis externa and, sometimes, in mycotic rhinitis synusitis in dolicephalic dogs. *Aspergillus fumigatus* is the more frequently involved agent as a consequence of its ability to grow at temperature higher than 40 °C. Otherwise, avian aspergillosis is a contagious life-threatening mycosis, which severely impacts with intensive breeding. In many countries, no systemic antifungal drugs are registered for use in birds, and resistance to antifungal drugs is very common in human *Aspergillus* isolates [149] as well as from birds. Cleaning and disinfection are basic tools in the management of such infections [150], and considering that a heavy colonized environment can expose human beings to professional diseases such as asthma, allergy, and “farmer lung” disease, the control of these molds in hen houses would be welcome. *A. flavus* and *A. fumigatus* are responsible for stonebrood, affecting both the brood and adult bees. These molds may develop on the body surface and/or affect capped and uncapped broods, making them similar to small stones. Behavioral changes are observed in adult bees, also. 

EOs has been checked against *A. fumigatus* cultured from a canine otologic specimen. *L. cubeba* scored strongly effective (MIC of 0.18 mg/mL), followed by *O. vulgare* (0.19 mg/mL), *R. officinalis* (0.29 mg/mL), and by *I. verum* (0.59 mg/mL) [36]. Moreover, the same agent isolated from fatal poultry aerosacculitis was shown to be likewise sensitive to *A. triphylla* and *C. citratus* with less active *L. cubeba* EO [8]. *A. terreus* isolated from canine otitis externa appeared less sensitive to EOs, except for *O. vulgare*, while *Aspergillus niger* from the same source was sensitive to *L. cubeba*, *O. vulgare*, *T. vulgaris,* and to *Ocimum basilicum* EOs [36]. 

*A. fumigatus* was reported to be strongly inhibited by *Leptospermum petersonii*, whose composition has been reported also [151], both in vitro and in vivo in a murine model [152]. 

The most effective EOs contain sabinene (*A. triphylla*) and citronellal (*A. triphylla L. petersonii*), neral, and geranial (*L. petersonii* and *C. citratus*). *L. cubeba* also is rich in high neral and geranial, but, although effective versus the otologic fungal isolate the EO, it was found to be less active against the poultry isolate, indicating a possible variability within the fungal species. The activity of *O. vulgare* can be attributed to its content in carvacrol that in an in vivo assay on poultry showed a MIC very similar to voriconazole when administered intratracheally. Conversely, eugenol and thymol displayed a moderate and negligible activity, respectively [153]. This finding is strongly corroborated by the marked antifungal activity of *O. syriacum* (containing more than 80% carvacrol) against three clinical specimens of *A. fumigatus* cultured from birds died from invasive aspergillosis [46]. 

Aspergilli responsible for stonebrood were tested and scored higly sensitive to several EOs (*I. verum*, *O. vulgare*, *T. vulgaris*, *L. cubeba*, *C. zeylanicum*, *C. flexuosus,* and *P. graveolens*) alone or in mixture [154].

### 3.4. Sporothrix brasiliensis

Sporotrichoses are worldwide mycoses caused by the dimorphic fungi belonging to genus *Sporothrix* [155]. The diseases affect humans and domestic animals, mainly cats and dogs, and they have a public health impact due to the zoonotic contamination potential through scratches and bites of cats. The emergent characteristics of feline and zoonotic sporotrichosis have been reported from several states in Brazil, where the species *Sporothrix brazilienesis* occurs, which is characterized by animal–animal or animal–human transmission [156,157]. In *Sporothrix* spp., the emergence of azole-resistant strains has been reported [158].

EOs and/or extracts from plants belonging to the *Lamiaceae* Family, mostly *O. vulgare* and *O. majorana*, are proven to be effective against this dimorphic fungus [158,159,160]. In particular, terpinen 4 ol and farnesol have shown MICs ranging from 87.9 to 1.5 μg/mL and from 0.003 to 0.2 μg/mL, respectively [161]. Terpinen-4-oil interferes with the fungal ergosterol [162,163] and has a synergistic action with conventional antifungal drugs [163], whilst c-terpinene hampers fungal growth, disrupting the structure of hyphae and reducing the number of conidia [164]. A recent in vivo study reports the successful administration of *O. majorana* (80 mg/kg for 8 days) to Wistar rats affected by experimental cutaneous sporothricosis by *S. brasiliensis*. The authors observed a prevention of fungal spread to internal organs and a decrease of fungal burden [162].

### 3.5. Pseudogymnoascus destructans

*Pseudogymnoascus destructans* is an emergent pathogen mold in Chiroptera that is responsible for White Nose Syndrome (WNS). The disease, discovered in caves near Albany (NY) in 2006 [165], in the last years has provoked a marked decline in the bats’ population, mostly in USA.

A drug treatment for WNS is not currently available [166]; furthermore, to avoid handling the bats, it is mandatory to dispose a therapy based on fine aerosol solution. So, in this view, EOs can be easily administered. 

*P. destructans* was inhibited by EOs obtained from cinnamon leaf and bark, citronella, and lemongrass [166] and by a terpenless Valencia orange oil that yielded an in vitro inhibition persisting up to 6 months as a consequence of a single application [167].

### 3.6. Ascosphaera apis

*Ascosphaera apis* is an entomopathogenic fungus responsible for chalkbrood, which is a mycosis of brood, mostly of *Apis mellifera*, whose occurrence is on the rise. Cool and humid climate, poor nutrition, stress, and genetic factors were considered as predisposing to the disease [168]. Larvae become infected by ingesting spores with food. The mold develops in the intestine, leading to the death of the larvae, which appear as mummies with a chalk appearence. At present, there is no treatment licensed to control it. A main issue is to avoid the possible presence of drug residues in the hive products; for this reason, different natural products have been screened [169].

Several EOs have been checked against *A. apis. C. zeylanicum* was successfully checked as not diluted [166], but when diluted, it showed an MIC higher than 5% [154], although *S. aromaticum* appeared quite effective [170]. This discrepancy could be attributed to the largest amount of *E*-Cinnamaldehyde (79.3%) in this latter with respect to *C. zeylanicum. L. cubeba* EO appeared very effective [154,170], probably for its richness in citral, which was present also in *C. flexuosus* EO [171]. EOs with high contents of geraniol were reported as active [163], and this finding was then corroborated by the antifungal activity of *P. graveolens* [154]. Carvacrol and thymol were shown to be effective [171]; however, such monoterpenoids seem to influence the insects’ behavior (i.e., the responses of bees to light) [172] and would not be a suitable choice in beekeeping. *S. aromaticum* yielded conflicting results [154,171]. The activity of some effective EOs was strongly enhanced when texted as a mixture such as *C. zeylanicum*, *C. flexuosus*, and *L. cubeba*, 0.02% each and *C. zeylanicum*, *C. flexuosus*, *P. graveolens,* and *L. cubeba*, 0.015% each, showing a synergistic action [154]. 

### 3.7. Nosema ceranae

Microsporidia are now classified into the Kingdom Fungi [173]. Among these organisms, *Nosema apis* and *Nosema ceranae* are responsible for bee nosemiasis, which is an infection that would reduce the honeybee population size and cause significant losses in hive production. Still, today, the only effective drug against *N. apis* is fumagillin, which is forbidden by most European countries [174]. This antibiotic is toxic to mammals and must be applied seasonally and with caution to avoid residues in honey. Furthermore, it seems to predispose bees to infection with *N. ceranae*, contributing to increasing the prevalence of this agent [175]. For these reasons, the study of alternative treatments is strongly required. 

EOs from *Laurus nobilis*, *O. vulgare*, *R. officinalis*, *Eucalyptus globulus*, and *C. zeylanicum* were orally administered [176] to bees naturally infected by *N. ceranae*, without any fungicidal effects, although they were attractive for bees. On the contrary, EO from *Cryptocarya alba* was very effective at a dose of 4 μg/bee, resulting in being more active with respect to its major components α terpineol, eucalyptol, and β phellandrene alone, which inhibited the organism at a dose of 20 μg/bee [177]. 

Table 2 reports the EOs that were shown to be active against mold and microsporidia strains of animal origin in in vitro and in vivo studies. 

### 3.8. Candida *spp.*

Yeasts are opportunistic pathogen organisms, most of which share with bacteria the ability to form biofilms. This form can colonize several medical devices, resulting in being less susceptible to antimycotic drugs.

Among the affections caused by yeasts, candidoses are a main medical issue, mostly in human medicine. Experimental murine models of oral or genitourinary candidoses are anyway available and are used both to study the pathogenesis and drugs response of the etiological agents. Then again, bovine mastitis and avian candidosis, together with candiduria and otitis in dogs and cats, are impacting affections on veterinary medicine.

*Candida albicans* is the most frequently involved yeast species, both in human and in veterinary medicine, and its ability to form a true mycelium in affected organs enhances the pathogenicity. However, other species such as *Candida tropicalis*, *Candida krusei*, *Candida glabrata*, *Candida parapsilosis,* and *Candida rugosa* are more and more involved in disease and are considered as emergent pathogens. Several strains, among these species, exhibit a resistance to azoles, with an increasing frequency [178]. 

Several studies carried out both in vitro and in vivo on animal models have been reported.

Suresh et al. [179] studied the antifungal in vitro effectiveness of *Santolina chamaecyparissus*, which yielded promising results, when administered to mice experimentally infected with *C. albicans.* This EO showed a synergistic effect with clotrimazole.

*C. albicans* was shown to be sensitive in vitro to *Mentha suaveolens*, which was able to heal an experimental murine vaginitis [180]. Geranium and geraniol seem to inhibit the switch to mycelial form at a 25 μg/mL concentration [111] and have improved an experimental vaginitis in mice, although the number of blastospores did not appear decreased [181]. Nerol, a monoterpene contained in some *Citrus* sp, was shown to be active in vitro and in vivo [182], being able to induce apoptosis [183]. This mechanism of action would be shared also by cinnamaldehyd, whose therapeutic effects in a mouse model of oropharyngeal candidiasis and vulvovaginal candidiasis have been recently reported [184].

*Anethum graveolens* seed EO was effective in vitro against *C. parapsilosis*, *C. krusei*, *C. tropicalis* and, at 2%, both in vitro and in vivo allowed to accelerate *C. albicans* 09-1555 clearance from vagina in experimentally infected mice when administered as prophylaxis and therapeutic treatments [185]. Oral experimental candidiasis was successfully treated in mice by the topical application of *M. alternifolia*. The dilution 12.5% was in vitro proven to exert a fungicidal, antibiofilm activity [186]. 

*C. albicans* was also checked with EOs obtained from *Artemisia judaica* and from *Tagetes lucida*, which did not yield any inhibiting effect, while a moderate activity was showed by *Artemisia dracunculus* [187]. *C. nepeta* was effective [96], and a very striking antifungal activity was showed by *T. numidicus* [97]. Ksouri et al. [188] successfully checked several bovine clinical isolates cultured from mastitis, with *Origanum floribundum* and *Thymus ciliatus.* These EOs are referred to be rich in phenols (thymol and carvacrol) precursors (p-cymene and g-terpinene) that allow their strong anticandidal action. These findings were recently confirmed by Ludwig et al. [189] who report a strong antifungal activity of carvacrol, thymol and, most of all, by cinnamaldehyde. 

Other studies in vitro concern different *Candida* species. In detail, *C. albicans* and *C. krusei* were shown to be resistant to EOs from *S. dolomitica* and *S. somalensis* [120]. *C. krusei* from poultry droppings was sensitive to *L. cubeba*, *T. vulgaris*, and *O. vulgare*, as well as *C. guilliermondii*. This finding is of interest considering that *C. krusei* would exhibit a marked resistance to common antimycotic drugs. Conversely, *C. albicans*, *C. tropicalis,* and *C. parapsilosis* from the same source were shown to be sensitive to *O. vulgare*, only [190]. This finding was partially corroborated by the proven sensitivity of *C. albicans* and *C. tropicalis*, isolated from otologic specimens, to this EO, as far as *R. officinalis* was shown to be active against this latter yeast species [36]. The efficacy of some EOs (*O. basilicum*, *O. vulgare*, *S. sclarea*, *T. vulgaris*, *I. verum*) was evaluated in different urine isolates of *Candida famata* and *C. albicans*, among which azole and fluconazole resistant strains. All fungi scored resistant to *S. sclarea* and exhibited different sensitivities to the other selected EOs. For instance, for *C. albicans*, MICs were ranging from more than 10 to 0.1 mg/mL. These findings confirm the different sensitivity to the same EO showed with the same species of yeast, suggesting the need of an individual checking for the in vitro efficacy of EOs, before to establish a treatment [20].

## 4. Oomycetes

Oomycetes or water molds are aquatic organisms, formerly classified as fungi, now classified in the kingdom *Protoctista* and related to heterokont, biflagellate, golden-brown algae. They differ from fungi because they neither synthetize ergosterol nor contain high amounts of chitin, not so being sensitive to conventional antimycotic drugs [191]. These organisms are considered as emerging threats to plants and animals [192] and will be considered in this review.

### 4.1. Saprolegnia spp.

Although more than 3000 EOs are known, less than 0.4% of them have been tested on fish parasites [193] and in particular on *Saprolegnia*. *Saprolegnia* spp. are oomycetes that cause serious problems to fish hatcheries. They are able to grow upon eggs and gills of fish, as a cotton-like mycelium, leading them to death. Saprolegniosis has been controlled by malachite green or formalin administration; however, with the ban of these products, which have a marked environmental toxicity, there is a demand for safe and effective natural products [194]. EOs have been checked against these organisms. *T. vulgaris* and *O. vulgare* were shown to be very active [195,196,197,198], which was probably due to high contents of carvacrol. These findings have been recently confirmed by Savadkouhi et al. [199], who reported the strong activity of a nanoessence of *Carum copticum*, which is rich in this compound. *O. majorana* [198] and *C. flexuosus* [195,198] appeared quite effective, and the same MICs were obtained also by *L. cubeba* and *C. bergamia* EOs [198]. However, this latter EO exerted a stronger anti-*Saprolegnia* efficacy (0.5% versus 2.5%) in another study [115]. The most inhibiting effect was probably due to the higher contents of linalool. Therefore, *C. bergamia* appeared more active against *Saprolegnia parasitica* when compared with several other *Citrus* spp, containing lower amounts of linalool and linalool acetate and lower concentrations of limonene, which were quite uneffective [198]. 

*Z. multiflora* and *Eucalyptus camaldulensis* EO yielded very low MICs against the oomycete, which were lower in respect to *Geranium herbarium* [200]. EO extracted from the bark of *Laureliopsis philipianna* also was reported to exert a strong activity against *Saprolegnia parasitica* and *Saprolegnia australis* [201].

A mixture of EOs from *T. vulgaris*, *S. officinalis*, *M. piperita,* and *E. globulus* yielded positive results [202], while a good efficacy was attributed also to *Atractyloides lancea* EO [203]. 

### 4.2. Pythium insidiosum

*Pythium insidiosum* is an oomycete causing invasive, ulcerative, proliferative, pyogranulomatous disease of the skin and subcutis in horses, but also in dogs and human patients. The organism occurs in tropical and subtropical areas and aquatic motile zoospores, which are able to penetrate small wounds and may cause vascular, ocular, gastrointestinal, and rarely systemic infections [204]. *P. insidiosum* has a low susceptibility to conventional antimycotic drugs [205], which is probably due to the absence of ergosterol in its cell membrane [206]. For these reasons, EOs have been employed for treatment. Compounds extracted from *Lamiaceae* have been checked. *O. vulgare* was shown to be strongly active, along with *O. majorana*, *M. piperita* [207], and *M. alternifolia*, too [205]. These EOs appeared very effective also in mixture. Namely, *O. vulgare* plus *M. piperita* showed a marked anti-*Pythium* activity [205]. These EOs were successfully used to treat experimental pythiosis in rabbit, alone, in mixture, and with immunotherapy [208]. On the other hand, carvacrol and thymol scored active against the organism and were efficaciously applied also associated with antibiotics and azoles [209], as well as the combination of *O. vulgare*, *M. alternifolia,* and *M. piperita* with itraconazole, while the mixture with terbinafine would yield an indifferent or antagonistic effect [210]. *M. alternifolia* 1% scored active in nanoemulsion, also [205].

## 5. Algae

### Prototheca spp.

*Prototheca* spp. are algae lacking chlorophyll. Some species within the genus are environmental saprophytes. Other species, among which *Prototheca zopfii*, *Prototheca wickerhamii,* and *Prototheca blaschkeae* are pathogenic organisms, responsible for epidemics of bovine mastitis, and, to a lesser extent, of canine and feline protothecosis. These latter are intestinal and/or systemic clinical forms and rare cutaneous disease, respectively. Bovine protothecosis economically impacts on milk yield, alongside the public health, due to the risk of zoonotic transmission, mostly to immunocompromised people. The human infection, although rare, is considered as emergent. The treatment of protothecosis consists of the administration of azoles (itra and fluconazole) and Amphotericin B; however, variate susceptibility drug patterns among the different *Prototheca* clinical isolates have been reported. So, more effective drugs are required to treat protothecosis [211].

There are few studies about the sensitivity of these algae to EOs. *C. paradisi* scored active at 0.75%, along with *T. vulgaris*, *L. cubeba*, and *O. vulgare* against *P. zopfii* and *P. blaschkeae. C. bergamia* showed the same MIC against *P. blaschkeae* [212] and was strongly effective against *P. wickerhamii* [213]. Otherwise, *T. vulgaris*, *O. majorana,* and to a lesser extent *O. vulgare* appeared active in another study [214], as well as *C. zeylanicum* and *S. aromaticum* [215]. Moreover, EOs from *M. piperita* and *Satureja hortensis* appeared effective in significantly reducing clinical signs of inflammation and fibrosis in vivo, in a murine model of cutaneous protothecosis induced by *P. zopfii* [216], although *M. piperita* and *S. montana* EOs did not yield any anti-alga in vitro effects [212]. On the other hand, the same authors yielded different MICs by the same EO (*T. vulgaris*) in two different studies [214,215], indicating some differences among the alga isolates and the EOs composition.

EOs that were shown to be active against yeast, oomycetes, and algae strains of animal origin in in vitro and in vivo studies are reported in Table 3.

## 6. Discussion and Conclusions

In the last years, some veterinarians have introduced EOs in the therapy of animals for different purposes, including to combat different pathogens. However, the studies available in the scientific literature regarding the employment of EOs in veterinary medicine are few in number.

Some studies reported the in vitro results obtaining assaying EOs versus some bacterial and fungal strains cultured from animal specimens. On the other hand, studies regarding in vivo effectiveness of EOs against bacterial and/or fungal infections in animals are very limited. 

There is strong evidence that a given EO can exhibit different antimicrobial behavior in relation to the tested pathogen. In fact, differences have been observed in relation to bacterial/fungal species, but also, within a same pathogen species, in relation to the strain. As well as executing an antibiotic susceptibility test before treating a bacterial infection in order to identify that the antibiotic really is active against an isolate, an aromatogram should be performed, assaying an isolate versus some EOs. After the most effective oils have been identified, a targeted phytotherapy with EOs against the pathogen could be possible.

As a result of the EOs toxicity, great attention must be used before treating companion and farm animals. For this reason, accurate investigation about the toxicity of a given EO in relation to animal species and the administration route are indispensable. 

EOs have been associated with hepatotoxicity, nephrotoxicity, changes in the blood vessels, reproductive toxicity, and oxidative stress that occur as a result of acute intoxication. However, this information is based on in vivo studies carried out mainly in rats and mice [217], whereas information about toxicity in domestic animals has not been elucidated.

As above referred, EOs are suitable for topic treatments when used as remedia; for these reasons, attention should be paid to possible adverse reactions such as photosensitivity, following the exposition to sunlight, which is frequently induced by several *Citrus* spp. The most frequently reported adverse effect is dermatitis. Lavender, peppermint, tea tree oil, and ylang-ylang are the most common essential oils responsible for side effects in human patients [218], so the EOs should be administered following the guidelines drawn up by an aromatherapist vet.

EOs could be employed in veterinary medicine also for disinfection purposes of different environments. In fact, they could be used in poultry farms and/or in hatcheries environment to control the spreading of bacteria but also to enhance the control of fungal spores’ burden, aiming to reduce the risk of infection for both animals and workers. Considering the importance of the farm hygiene to prevent the introduction and spreading of pathogens, a similar use could be applied in farms of other animal species.

Domestic environment could be disinfected through EOs application, too. A herbal mixture composed of *L. cubeba* (1%), *I. verum*, *F.*, and *P. graveolens* (0.5% each) EOs in 95% ethanol showed a good antifungal activity against arthrospores present in infected hairs. These findings allowed applying the mixture as an adjuvant in the environmental control of feline microsporiasis [219].

In conclusion, knowledge about positive and negative properties of EOs needs to be improved involving more disciplines, such as microbiology, parasitology, toxicology, and pharmacology, performing investigations focused on the veterinary medicine.

## Figures and Tables

**Table 1:** Essential oils were shown to be active against bacterial strains of animal origin in in vitro and in vivo studies.

Bacterial Pathogen	Study	Animal Source	Active Essential Oils	References
*Escherichia coli*	In vitro	Poultry	*Cinnamomum zeylanicum*	[8]
			*Syzigium aromaticum*	[8]
			*Litsea cubeba*	[8]
			*Cymbopogon citratus*	[8,13]
			*Mentha piperita*	[8,14]
			*Ocimum basilicum*	[8]
			*Pelargonium graveolens*	[8,17,18]
	In vitro	Dog	*Origanum vulgare*	[20]
			*Thymus vulgaris*	[20]
			*Illicium verum*	[20]
			*ocimum basilicum*	[20]
			*Salvia sclarea*	[20]
*Salmonella enterica* serov Enteritidis and Typhimurium	In vitro	Poultry	*Cinnamomum zeylanicum*	[23]
			*Syzigium aromaticum*	[23]
*Salmonella enterica* serov. Enteritidis	In vivo	Poultry	EOA1, EOA2 *	[27]
*Pseudomonas aeruginosa*	In vitro	Dog	*Salvia sclarea*	[36]
			*Ocimum basilicum*	[36]
			*Rosmarinus officinals*	[36]
*Pseudomonas* spp.	In vitro	Fish	*Cinnamomum zeylanicum*	[40]
			*Lavandula angustifolia*	[40]
			*Pinus montana*	[40]
			*Pinus sylvestris*	[40]
			*Mentha piperita*	[40]
			*Foeniculum vulgare*	[40]
			*Satureja hortensis*	[40]
			*Origanum vulgare*	[40]
			*Pimpinella anisum*	[40]
			*Rosmarinus officinalis*	[40]
			*Salvia officinalis*	[40]
			*Abies alba*	[40]
			*Citrus aurantium* var. *dulce*	[40]
			*Citrus sinensis*	[40]
			*Cymbopogon nardus*	[40]
			*Mentha spicata* var. *crispa*	[40]
			*Thymus vulgaris*	[40]
			*Thymus serpyllum*	[40]
			*Ocimum basilicum*	[40]
			*Coriandrum sativum*	[40]
			*Carum carvi*	[40]
*Campylobacter jejuni*	In vitro	Poultry	*Leptospermum* sp.	[45]
			*Melaleuca alternifolia*	[45]
			*Backhousia citriodora*	[45]
*Staphylococcus* spp.	In vitro	Dog	*Cinnamomum zeylanicum*	[50,57]
			*Commiphora myrrha*	[50]
			*Helichrysum pandurifolium*	[55,56]
			*Helichrysum trilineatum*	[55,56]
			*Helichrysum araxinum*	[55,56]
			*Satureja montana*	[57]
			*Melissa officinalis*	[57]
			*Leptospermum scoparium*	[57]
			*Thymus vulgaris*	[36,50]
			*Origanum vulgare*	[36,50]
*Staphylococcus* spp.	In vivo	Cattle	*Thymus vulgaris*	[63]
			*Lavandula angustifolia*	[63]
*Streptococcus iniae*	In vivo/in vitro	Fish	*Cinnamomum verum*	[69]
	In vivo	Fish	*Syzigium aromaticum*	[70]
*Streptococcus suis*	In vitro	Swine	*Thymus vulgaris*	[74]
			*Origanum vulgare*	[74]
*Enterococcus* spp.	In vitro	Dog/Cat	*Thymus vulgaris*	[20]
			*Origanum vulgare*	[20]
*Mycobacterium* spp (NTM)	In vitro	Not specified	*Zingiber officinale*	[86]
*Mycobacterium avium*	In vitro	Cattle	*Origanum vulgare*	[89,90]
*paratuberculosis*			*Cinnamomum* sp.	[89,90]

* Legend—EOA1 (thymol, carvacrol, benzoic acid, butyric acid, and excipients such as silicon dioxide and palm oil); EOA2 (cinnamylaldehyde, caproic acid, benzoic acid, butyric acid, and the excipients silicon dioxide and palm oil).

**Table 2:** Essential oils that were shown to be active against mold and microsporidia strains of animal origin in in vitro and in vivo studies.

Fungal Pathogen	Study	Animal Source	Active Essential Oils	References
*Microsporum canis*	In vitro	Feline, dog, horse	*Thymus vulgaris*	[95]
			*Satureja montana*	[95]
	In vitro	Feline	*Thymus numidicus*	[97]
	In vitro	Feline	*Cymbopogon nardus*	[112]
	In vitro	Feline, canine	*Coriandrum sativum*	[113]
	In vitro	Feline	*Helichrysum pandurifolium*	[55]
	In vitro	Feline	*Litsea cubeba*	[100]
			*Origanum vulgare*	[100]
			*Thymus serpyllum*	[100]
	In vitro/in vivo *	Feline	Mixture: *T. serpyllum* 2%	[98,129]
			*Origanum vulgare* 5%, *Rosmarinus officinalis* 5%	
*Microsporum gypseum*	In vitro	Dog, horse	*Thymus vulgaris*	[95]
			*Satureja montana*	[95]
			*Calamintha nepeta*	[95]
	In vitro	Dog	*Thymus numidicus*	[97]
	In vitro	Dog	*Litsea cubeba*	[100]
			*Origanum vulgare*	[100]
			*Thymus serpyllum*	[100]
	In vitro/in vivo **	Stock culture strain	*Japanese cypress oil*	[130]
	In vitro/in vivo **	Stock culture strain	*Cymbopogon martini*	[133]
			*Chenopodium ambrosoides*	[132]
*Trichophyton mentagrophytes*	In vitro	Feline	*Clinopodium nepeta* var *glandulosum*	[96]
	In vitro	Feline	*Thymus numidicus*	[97]
	In vitro	Feline	*Litsea cubeba* *Thymus serpyllum*	[100]
	In vitro/in vivo ***	Ovine	*Mixture: Thymus serpyllum 2%Origanum vulgare* 5%, *Rosmarinus officinalis* 5%	[99]
*Trichophyton erinacei*	In vitro	Dog	*Litsea cubeba*	[100]
*Trichophyton equinum*	In vivo ****	Equine	*Melaleuca alternifolia*	[118]
	In vitro	Equine	*Melaleuca alternifolia*	[119]
*Malassezia pachydermatis*	In vitro	Dog	*Melaleuca alternifolia*	[137,138]
	In vitro	Dog	*Zataria multiflora*	[140]
	In vitro	Dog	*Thymus* spp. *Satureja montana*	[140,141,142]
	In vitro	Dog	*Origanum vulgare*	[50,143]
	In vitro	Dog	*Cymbopogon spp*	[141,142,143]
	In vitro	Dog	*Citrus limon*	[143]
		Dog	*Mentha piperita*	[142]
	In vitro	Dog	*Cinnamomum sp*	[50,143,144]
	In vitro	Dog	*Aloysia triphylla*	[50]
	In vivo §	Dog	*Chamaecyparis obtusa*	[145]
	In vitro/in vivo §	Dog	Mixture: *Citrus aurantium* 1%, *Lavandula* *officinalis* 1%, *Origanum vulgare* 0.5%, *Origanum majorana* 5%, *Mentha piperita* 0.5%, *Helichrysum italicum* 0.5%	[147]
	In vitro/in vivo §	Dog	Mixtures: 1. *Citrus limon* 1% *Rosmarinus officinalis* 1%, *Salvia sclarea* 0.5%, *Anthemis nobilis* 0.5% 2. *Citrus paradisi* 0.5%, *S. sclarea* 0,5%, *Ocimum basilicum* 0,5%, *R. officinalis* 1% 3. *S. sclarea* 1%, *Lavandula hybrida* 1%, *R. officinalis*	[148]
*Aspergillus fumigatus*	In vitro	Dog	*Litsea cubeba*	[36]
			*Origanum vulgare*	[36]
			*Illicium verum*	[36]
			*Rosmarinus officinals*	[36]
	In vitro	Poultry	*Cymbopogon citratus*	[8]
			*Aloysia triphylla*	[8]
	In vitro	Birds	*Origanum syriacum*	[46]
	In vitro/in vivo °	-	*Leptospermum petersonii*	[152]
*Aspergillus flavus*	In vitro	Bee stonebrood	*I. verum*, *O. vulgare*, *L. cubeba*, *Cynnamomum zeylanicum*, *Cymbopogon flexuosus*, *Pelargonium graveolens* Mixtures: 1 *L. cubeba*, *C. zeylanicum*, *Cymbopogon flexuosus* 0.02% each 2 *L. cubeba*, *C. zeylanicum*, *P. graveolens*, *C. flexuosus* 0.015% each	[154]
*Sporothrix brasiliensis*	In vitro	Stock	*Origanum majorana*	[161]
			*Origanum vulgare*	[161]
	In vivo $	Feline, canine	*O. majorana*	[162]
*Pseudogymnoascus destructans*	In vitro	Bat	Cinnamon leaf and bark, citronella, lemongrass	[166]
	In vitro	Bat	Terpenless Valencia orange	[167]
*Ascosphaera apis*	In vitro	Bee chalkbrood	*Syzygium aromaticum*	[170]
			*Litsea cubeba*	[154,170]
			*Cymbopogon flexuosus*	[171]
			Mixtures: 1 *L. cubeba*, *Cinnamomum zeylanicum*, *Cymbopogon flexuosus* 0.02% each 2 *Litsea cubeba*, *Cinnamomum zeylanicum*, *Pelargonium graveolens*, *Cymbopogon flexuosus* 0.015% each	[154]
*Nosema ceranae*	In vivo ^	Bee	*Cryptocarya alba*	[177]

Legend—* in vivo administration on cats; ** in vivo administration on Guinea pigs; *** in vivo administration on sheep; **** in vivo administration on horses; § in vivo administration on dogs; ° in vivo administration on mouse; $ in vivo administration on rats; ^ in vivo administration on bees.

**Table 3:** Essential oils that were shown to be active against yeast, oomycetes, and algae strains of animal origin in in vitro and in vivo studies.

Pathogen	Study (*)	Animal Source	Active Essential Oils	References
*Candida albicans*	In vitro/in vivo	Stock culture strain	*Santolina chamaecyparissus*	[179]
	In vitro/in vivo	Stock culture strain	*Mentha suaveolens*	[180]
	In vitro/in vivo	Stock culture strain	*Geranium* sp	[111,181]
	In vitro/in vivo	Stock culture strain	*Citrus* sp	[182,183]
	In vitro/in vivo	Stock culture strain	cinnamaldehyd	[184]
	In vitro/in vivo	Stock culture strain	*Anethum graveolens*	[185]
	In vitro	Birds	*Clinopodium nepeta*	[96]
	In vitro	Birds	*Thymus numidicus*	[97]
	In vitro	Bovine	*Origanum floribundum*	[188]
			*Thymus ciliatus*	[188]
	In vitro	Poultry	*Origanum vulgare*	[190]
*Candida rugosa*	In vitro	Animals **	cinnamaldehyd	[189]
*Candida tropicalis*	In vitro	Dog	*Origanum vulgare*	[36]
	In vitro	Poultry	*Origanum vulgare*	[190]
	In vitro	Dog	*Salvia sclarea*	[20]
	In vitro	Dog	*Origanum vulgare*	[36]
			*Rosmarinus officinalis*	[36]
*Candida parapsilosis*	In vitro	Poultry	*Origanum vulgare*	[190]
*Candida guilliermondii*	In vitro	Poultry	*Litsea cubeba*	[190]
			*Origanum vulgare*	[190]
*Candida krusei*	In vitro	Poultry	*Thymus vulgaris*	[190]
*Saprolegnia* spp	In vitro	Fish	*Thymus vulgaris*	[195,196,197,198]
			*Origanum vulgare*	[195,196,197,198]
	In vitro	Fish	*Carum copticum*	[199]
	In vitro	Fish	*Origanum majorana*	[198]
	In vitro	Fish	*Cymbopogon flexuosus*	[195,198]
	In vitro	Fish	*Citrus bergamia*	[115]
	In vitro	Fish	*Zataria multiflora*	[50,143,144]
	In vivo °	Fish	*Eucalyptus camaldulensis*	[200]
	In vitro	Fish	*Laureliopsis philipianna*	[201]
	In vitro	Fish	Mixture: *Thymus vulgaris*, *Salvia*	[202]
			*officinalis*, *Mentha piperita Eucalyptus globulus*	
	In vitro	Fish	*Atractyloides lancea*	[203]
*Pythium insidiosum*	In vitro	Horse and dog	*Melaleuca alternifolia*	[205]
	In vitro	Horse	*Origanum vulgare*	[205,208]
			*Origanum majorana*	[205,208]
			*Mentha piperita*	[205,208]
	In vivo	Not specified	*Origanum vulgare + Mentha piperita*	[210]
*Prototheca zopfii*	In vitro	Bovine	*Thymus vulgaris*	[212]
			*Litsea cubeba*	[212]
			*Origanum vulgare*	[212]
	In vitro	Bovine	*Thymus vulgaris*	[214]
			*Origanum vulgare*	[214]
	In vitro	Bovine	*Cinnamomum zeylanicum*	[215]
			*Syzygium aromaticum*	[215]
	In vivo	Bovine	*Mentha piperita*	[216]
			*Satureja hortensis*	[216]
*Prototheca blaschkeae*	In vitro	Bovine	*Thymus vulgaris*	[212]
			*Litsea cubeba*	[212]
			*Origanum vulgare*	[212]
			*Citrus bergamia*	[212]
*Prototheca wickerhamii*	In vitro	Bovine	*Citrus bergamia*	[213]

(*) Legend—all the in vivo studies were carried out on murine models, except for Pythium (rabbits) (**) Cows, dogs, horse coati, hawk (°) Rainbow trout eggs.
